# Insight into the PTP1B Inhibitory Activity of Arylbenzofurans: An In Vitro and In Silico Study

**DOI:** 10.3390/molecules24162893

**Published:** 2019-08-09

**Authors:** Srijan Shrestha, Su Hui Seong, Seul Gi Park, Byung Sun Min, Hyun Ah Jung, Jae Sue Choi

**Affiliations:** 1 Department of Food and Life Science, Pukyong National University, Busan 48513, Korea; 2 Discipline of Pharmacology, School of Medicine, Faculty of Health Sciences, The University of Adelaide, Adelaide 5005, South Australia, Australia; 3 Department of Food Science and Human Nutrition, Chonbuk National University, Jeonju 54896, Korea; 4 College of Pharmacy, Drug Research and Development Center, Catholic University of Daegu, Gyeongbuk 38430, Korea

**Keywords:** 2-arylbenzofurans, PTP1B, T2DM, in silico studies

## Abstract

Protein tyrosine phosphatase 1B (PTP1B) plays a specific role as a negative regulator of insulin signaling pathways and is a validated therapeutic target for Type 2 diabetes. Previously, arylbenzofurans were reported to have inhibitory activity against PTP1B. However, detailed investigation regarding their structure activity relationship (SAR) has not been elucidated. The main aim of this work was to investigate the PTP1B inhibitory activity of 2-arylbenzofuran analogs (sanggenofuran A (SA), mulberrofuran D2 (MD2), mulberrofuran D (MD), morusalfuran B (MB), mulberrofuran H (MH)) isolated from the root bark of *Morus alba.* All compounds demonstrated potent inhibitory activity with IC_50_ values ranging from 3.11 to 53.47 µM. Among the tested compounds, MD2 showed the strongest activity (IC_50_, 3.11 µM), followed by MD and MB, while SA and MH demonstrated the lowest activity. Lineweaver-Burk and Dixon plots were used for the determination of inhibition type whereas ligand and receptor interactions were investigated in modeled complexes via molecular docking. Our study clearly supports 2-arylbenzofuran analogs as a promising class of PTP1B inhibitors and illustrates the key positions responsible for the inhibitory activity, their correlation, the effect of prenyl/geranyl groups, and the influence of resorcinol scaffold, which can be further explored in-depth to develop therapeutic agents against T2DM.

## 1. Introduction

With the rapid increase in the incidence of type 2 diabetes mellitus (T2DM) in the world, there is a great need for new therapeutic agents for T2DM prevention and treatment as current therapies are limited and often ineffective. The genetically-susceptible obese population is prone to insulin resistance and impaired glucose tolerance (IGT). Furthermore, pancreatic cells fail to compensate for insulin resistance due to gluco- and lipotoxicity, which lead to T2DM [1]. Protein tyrosine phosphatase 1B (PTP1B) is a negative regulator of insulin receptor phosphorylation and signaling and can associate with dephosphorylated activated insulin receptor (IR) or insulin receptor substrates (IRS). Further, PTP1B knockout mice have insulin sensitivity and are resistant to obesity on a high-fat diet [2,3]. These results validate PTP1B as a key negative regulator of insulin signal transduction and as an attractive target for T2DM treatment.

PTP1B (50 kDa) consists of 435 amino acid residues with an N-terminal catalytic region (1–300), a regulatory region (300–400), and a C-terminal membrane localization region (400–435). The protein consists of eight α-helices and eleven β-strands. The R loop (Val113-Ser118), lysine loop (Leu119-Cys121), WPD loop (Thr177-Pro185), S loop (Ser201-Gly209), Q loop (Ile261-Gln262), α3 helix (Glu186-Glu200), α6 helix (Ala264-Ile281), and α7 helix (Val287-Ser295) regions play critical roles in the dephosphorylation of phosphotyrosine [4].

The heterocyclic compound 2-arylbenzofurans consists of fused benzene and furan rings along with different substituted groups (complex structures) at the C-2 position. These compounds show a wide range of pharmacological properties including anticancer [5], antibacterial [6], antimicrobial [7], antifungal [8], anticonvulsant [9], anti-inflammatory [10,11], and inhibition of Aβ fibril formation [12] activity. Extensive research has been done by synthetic chemists to exploit the biological activity of 2-arylbenzofurans. However, very little is known about safe natural benzofurans, and considerable natural product research has been conducted to characterize the multi-functional activity of natural compounds that also offer fewer side effects compared to modern synthetic treatments.

The *Morus* (Moraceae) genus consists of 10 to 16 different species of deciduous trees called mulberries that can be found in the wild and under cultivation in Asia, Africa, and America. Traditionally, *Morus alba* root bark has been used as an antidiabetic, diuretic, expectorant, laxative agent, and used to treat arthritis, rheumatism, and various stomach disorders [13,14,15]. Previously, we reported the PTP1B inhibitory and anti-Alzheimer activities of compounds isolated from the root bark of *Morus* [16,17,18]. Interestingly, mulberrofuran G (MG), which consists of a 2-arylbenzofuran moiety, showed the most potent inhibitory activity [16]. In addition, Paudel et al. argued that mulberrofuran D2 (MD2) as a promising drug candidate looking into its potency, ADME and drug-likeness [18]. As such, we directed our search for the isolation of MG analogs to establish the structure activity relationships (SARs). Herein, we have isolated five compounds, sanggenofuran A (SA), MD2, mulberrofuran D (MD), morusalfuran B (MB), and mulberrofuran H (MH), and evaluated their activity via PTP1B inhibitory assays in an effort to understand the molecular mechanism of these compounds via kinetics and docking studies.

## 2. Results

### 2.1. PTP1B Inhibitory Assays

**Figure 1:**
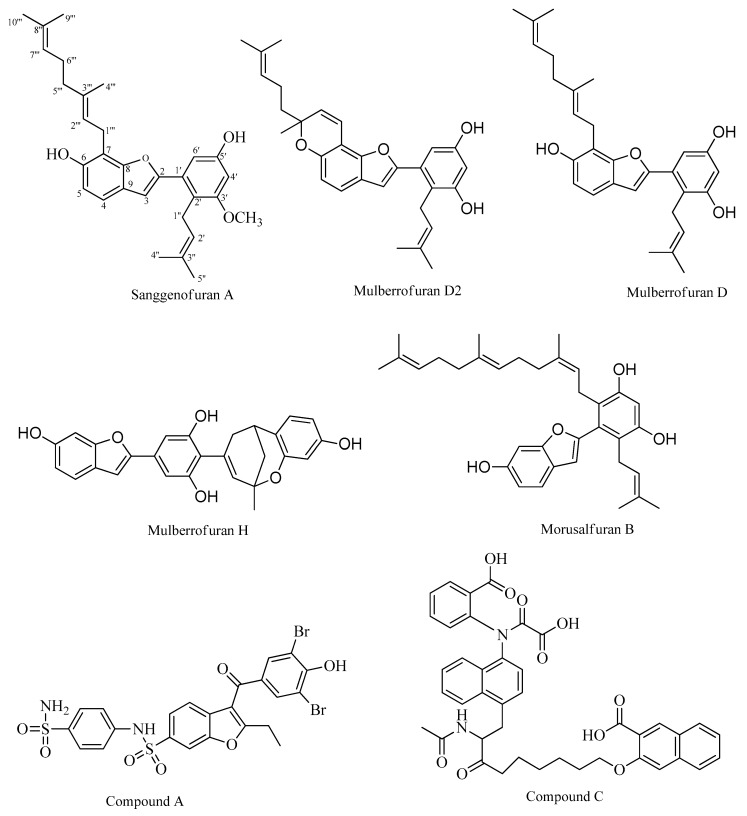
Structures of five 2-arylbenzofurans, one allosteric inhibitor (compound **A**), and one catalytic inhibitor (compound **C**) selected for our study.

All compounds inhibited hydrolysis of the p-nitrophenyl phosphate (pNPP) substrate catalyzed by PTP1B in a dose-dependent manner with IC_50_ values ranging from 3.11 to 53.47 µM (Table 1). Among the tested compounds (Figure 1), MD2 showed pronounced inhibitory activity with an IC_50_ value of 3.11 ± 0.10 μM, followed by MD, MB, SA, and MH, with IC_50_ values of 11.61 ± 0.19 μM, 12.00 ± 0.75 μM, 31.85 ± 2.98 μM, and 53. 47 ± 12.5 μM, respectively. A known PTP1B inhibitor, ursolic acid (IC_50_; 7.47 μM), was used as a positive control.

### 2.2. Kinetic Studies

**Figure 2:**
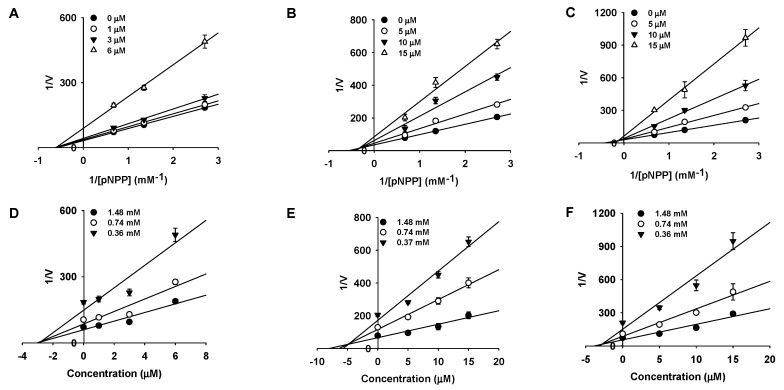
Lineweaver-Burk and Dixon plots for PTP1B inhibition of mulberrofuran D2 (MD2) (**A** and **D**), mulberrofuran D (MD) (**B** and **E**), and morusalfuran B (MB) (**C** and **F**), respectively.

Since three compounds (MD2, MD, and MB) demonstrated pronounced activity, they were subjected to kinetic studies at various compound concentrations and *p*NPP to understand their inhibition type. Here, we employed Lineweaver-Burk and Dixon plots to determine the inhibition type and inhibition constant (*K*_i_ value) for each compound (Figure 2, Table 1). MD2 showed non-competitive inhibition as revealed by the Lineweaver-Burk plot (*K*_m_ value constant but the *V*_max_ value changed) with a *K*_i_ value of 2.63 μM. However, MD and MB showed mixed-type inhibition (*V*_max_ value and the *K*_m_ value varies) with *K*_i_ values of 4.79 and 2.84 μM, respectively.

### 2.3. In Silico Docking Studies

**Figure 3:**
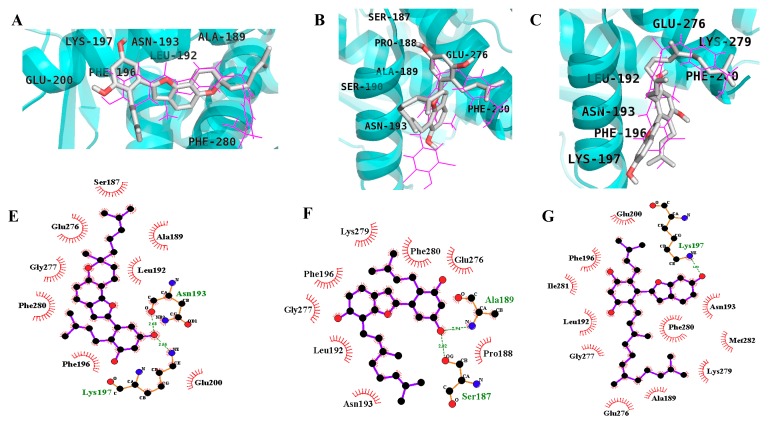
Binding mode for the PTP1B allosteric site with reported inhibitor compound **A** (pink line) by MD2 (**A**), MD (**B**), and MB (**C**). 2-D ligand interaction diagram of PTP1B allosteric inhibition by MD2 (**E**), MD (**F**), and MB (**G**). Dashed green lines indicate H-bonds. Carbon is in black, nitrogen is in blue, and oxygen is in red.

**Figure 4:**
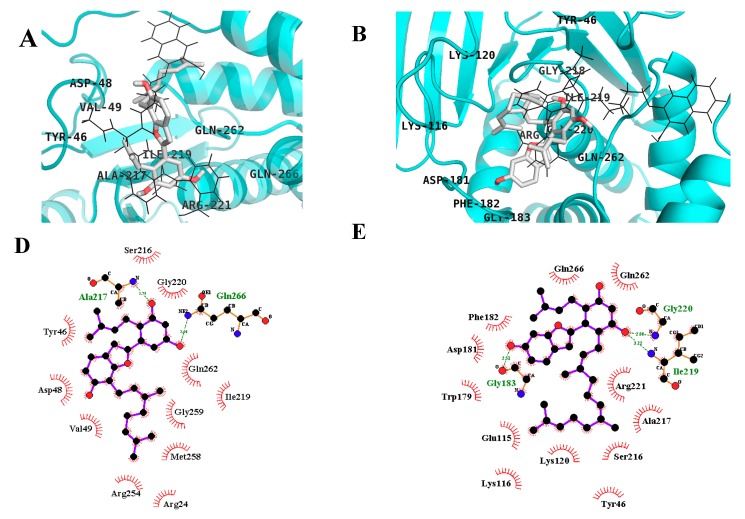
Binding mode for the PTP1B active site with reported inhibitor compound **C** (black line) by MD (**A**) and MB (**B**). 2-D ligand interaction diagram of PTP1B catalytic inhibition by MD (**D**) and MB (**E**). Dashed green lines indicate H-bonds. Carbon is in black, nitrogen is in blue, and oxygen is in red.

The compounds were subjected to in silico studies to confirm and understand the binding modes of the different inhibitors. To validate and optimize the docking procedure, we re-docked a native co-ligand, compound **A**, (3-(3,5-dibromo-4-hydroxy-benzoyl)-2-ethyl-benzofuran-6-sulfonic acid (4-sulfamoyl-phenyl)-amide) into the allosteric site of PTP1B (PDB ID: 1T49) [19]. Wiesmann et al. synthesized the compound **A**, and crystallographic analysis was done in order to validate it as an allosteric inhibitor of PTP1B (IC_50_, 22 μM) that binds to a novel site located ∼20 Å from the catalytic site [19]. The root mean square deviation (RMSD) value was obtained by comparing the best pose generated in AutoDock 4.2 with our docking protocol and the co-ligand (0.56 Å). This validated method was used to investigate the binding pose of the selected compounds, with the most stable docking poses shown (Figure 3 and Figure 4, and Table 2). Figure 3A,E shows a complete view of the MD2 docking pose to the allosteric site of PTP1B. The lowest energy docking configuration of MD2 (−9.51 kcal/mol) is in good agreement with experimental data and places MD2 in the proximity of Lys197, Asn193, Phe280, Phe196, Leu192, Ala189, Glu200, Ser187, Glu276, and Gly277. MD2 formed two hydrogen bonds with Asn193 and Lys197 via the C5′ -OH (resorcinol group) and the remaining mentioned residues were involved in hydrophobic interactions. Interestingly, all the other compounds demonstrated good binding affinity in the catalytic and allosteric binding sites while using a 126 × 126 × 126 grid box around the enzyme (PDB, 1t49), which is consistent with our kinetic results. 

As shown in Figure 4A,D, MD displayed two H-bonds with Gln266 and Ala217 via C3′ and C5′ -OH, respectively, and hydrophobic interactions with Asp48, Tyr46, Val49, Met258, Arg254, Gly259, Gln262, Arg24, Ile219, Gly220, and Ser216 with a binding energy of −6.71 kcal/mol at the catalytic site whereas the allosteric site (−7.93 kcal/mol) showed two H-bonds with Ala189 and Ser187 via C5′ -OH and hydrophobic interaction with Asn193, Phe196, Gly277, Leu192, Lys279, Phe280, Glu276, and Pro188 (Figure 3B,F). Similarly, MB (C5′-OH) interacted with Gly220 and Ile219 via H-bonds in the catalytic domain of PTP1B. Further, the C6 -OH of MB showed additional H-bond interaction with Gly183 and the hydrophobic interactions included Phe182, Gln266, Gln262, Asp181, Trp179, Glu115, Lys116, Lys120, Tyr46, Ser216, Ala217, and Arg221 (Figure 3C,G and Figure 4B,E).

## 3. Discussion

The rapid increase in people at risk for diabetes is a major public health concern. T2DM, which occurs in a majority of diabetes cases, has now been reported to occur in children (only adults were vulnerable previously) [20]. PTP1B has been the subject of interest in recent years as many reports firmly favor it as a promising target for T2DM as its expression in muscle and adipose tissue correlate with the degree of insulin resistance and the attenuation of leptin signaling pathways [2,3,21,22]. Therefore, targeting and developing novel PTP1B inhibitors from natural sources may lead to an effective therapeutic agent without the side effects (gastrointestinal stress, weight gain, peripheral edema, headache, hypotension) associated with synthetic drugs including α-glucosidase inhibitors, insulin secretagogues, biguanides, and thiazolidinediones [23].

Benzofurans are a popular class of compounds that have been explored by synthetic chemists for decades for use in various biological functions. Some of the clinically-approved benzofurans include dronedarone, amiodarone (antiarrhythmic agent), saprisartan (hypertension and heart failure), vilazodone (antidepressant), and 6-(2-aminopropyl) benzofuran (psychoactive drugs), to name a few. However, little information regarding naturally-occurring arylbenzofurans can be found in the literature, especially in the case of PTP1B inhibitors. Recently, we reported the anti-Alzheimer’s and anti-diabetic potential of 2-arylbenzofurans from *M. alba* [16,17]. Looking into its potential, we further explored the chloroform fraction of *M. alba* to identify additional structural analogs with potent PTP1B inhibitory activity to elucidate SARs.

Among the tested compounds, MD2, MD, and MB demonstrated potent inhibitory activity, whereas SA and MH displayed moderate inhibitory activity against PTP1B. Structurally, MD2 possesses a pyrone ring at the α-position of the benzene ring in 2-arylbenzofuran, which might be the reason behind its pronounced activity. No significant difference in activity was found while comparing MD and MB (which differ only in the prenyl/geranyl moiety position), suggesting that the prenyl/geranyl group position might not be important in regards to PTP1B activity and that inhibitor structure is tolerable of certain scaffold variations. Surprisingly, upon replacement of the C3′-OH group with an -OCH_3_ group, activity was significantly reduced (>3 times), suggesting the importance of the resorcinol scaffold for optimum activity (SA vs. MD). In addition, the activity of MH was almost five times less potent than MD/MB, which signifies the importance of the prenyl/geranyl group and suggests that the presence of a bulkier group in the C4′ position may hinder reactivity of the ortho OH-groups, which are essential for compound activity. Seong and Zhang also suggested similar findings, where they showed that the resorcinol scaffold and prenyl moiety play a significant role in the inhibitory activity [17,24]. Furthermore, our enzyme kinetic studies revealed MD2 as a non-competitive inhibitor and MD and MB as a mixed type inhibitor by comparing the obtained experimental data with different compound concentrations and *p*NPP in Lineweaver-Burk plots (1/V vs. 1/S).

With the identification of catalytic and allosteric sites in PTP1B using X-ray crystallographic structure, various inhibitors have been discovered and characterized, including their molecular dynamics, pharmacophore, helix modeling, and energy analysis. Wiesmann et al. demonstrated the selective inhibition of compound **A**, (3-(3,5-dibromo-4-hydroxy-benzoyl)-2-ethyl-benzofuran-6-sulfonic acid (4-sulfamoyl-phenyl)-amide) against PTP1B (PDB ID: 1T49) [19]. We used the same (1T49) crystallographic structure for our analysis. All compounds were docked using a 126 × 126 × 126 grid box size to determine the binding pose of the compounds. MD2 showed interaction with the important Asn193, Phe196, and Phe280 binding residues via H-bond and hydrophobic bonds, respectively, which are important for inhibitory activity as suggested by Wiesmann [19]. The ligand complex structure revealed that MD2 binds to a site formed by helices α3 and α6. The benzofuran core of MD2 sits in a hydrophobic pocket formed by the side chains of Leu192, Phe196, and Phe280, similar to compound **A** (used as a selective allosteric inhibitor), which explains its potent activity. Surprisingly, all the other docked compounds displayed binding in both the catalytic and allosteric sites, which is consistent with our experimental kinetic studies. MD binds to the catalytic P-loop through H-bonds at residues Gln266 and Ala217 and hydrophobic interactions, but no interaction with Cys215 was observed as seen in the case of compound **C** (selective catalytic inhibitor) as mentioned by Szczepankiewicz [25]. However, MD displayed interactions with important catalytic binding residues, Tyr46, Gln262, and Ser216. As in the case of the allosteric binding site, MD showed interactions with the important residues Asn193, Phe196, Phe280, and Glu276 via hydrophobic bonds but the binding pose was different from MD2. These interactions and conformations explain the potency of this compound.

As shown in Figure 4E, MB is sandwiched between Tyr46 and Phe182 and forms hydrophobic bonds with the side chain of active site residues (Ser216 and Arg221). Additionally, MB showed interactions with important binding residues of the allosteric site and was almost superimposed with compound **A** (Figure 3C). On the contrary, the -OH group of the resorcinol scaffold did not form any H-bonds with the allosteric residues. Overall, the resorcinol scaffold seems to be the most important scaffold in this class of compounds as revealed by docking studies due to the involvement of hydrogen bonding.

The allosteric site of PTP1B is susceptible to binding small molecules, consists of more hydrophobic residues, and is not well conserved, which gives an opportunity to avoid the problems associated with catalytic inhibitors. So, extensive research has been directed towards the discovery of allosteric inhibitors. The selective allosteric inhibitor compound **A** **[19]** and our potent compound MD2 share the same benzofuran parent moiety and is 7-times more potent than A. Thus, MD2 (lead compound) and other structural analogs (2-arylbenzofurans) with PTP1B can be further explored with advanced X-ray crystallography, cell-based assays, and in vivo experiments in order to confirm their functionalities and contribute to develop a therapeutic agent for T2DM.

## 4. Materials and Methods

### 4.1. Chemicals and Reagents

Ethylenediaminetetraacetic acid, *p*-nitrophenyl phosphate (*p*NPP), and dimethylsulfoxide were purchased from Sigma-Aldrich Co. (St. Louis, MO, USA). Protein tyrosine phosphatase 1B (PTP1B, human recombinant) and dithiothreitol (DTT) were purchased from Biomol^®^ International LP (Plymouth Meeting, PA, USA) and Bio-Rad Laboratories (Hercules, CA, USA), respectively. All chemicals and solvents used in the isolation and enzyme assays were of reagent grade and were purchased from commercial sources. TLC were performed on a precoated Merck Kiesel gel 60 F_254_ plates (20 × 20 cm, 0.25 mm) and RP-18 F_254S_ plates (5 × 10 cm) (Merck, Darmstadt, Germany).

### 4.2. Plant Materials

The root bark of *M. alba* was collected from Ulsan province (Republic of Korea) in 2016, and authenticated by professor Byung-Sun Min, College of Pharmacy, Daegu Catholic University, Republic of Korea. The voucher specimen was deposited at the Herbarium of the College of Pharmacy, Daegu Catholic University.

### 4.3. Extraction and Isolation

A MeOH extract (995.5 g) of *M. alba* root bark was suspended in distilled water (dH_2_O) and successively partitioned with *n*-hexane, CHCl_3_, and EtOAc. The CHCl_3_ fraction (215.2 g) was subjected to silica gel column chromatography (CC) using a CHCl_3_:MeOH (1:0 to 0:1, gradient, *v/v*) solvent system, which afforded 19 subfractions (F1–F19).

Compound **1** (33 mg) was isolated from subfraction F2 via silica gel CC with a *n*-hexane–acetone (100:0 to 0:100, gradient, *v/v*) solvent system and reversed-phase C_18_ (RP-C_18_) silica gel CC with an acetonitrile–H_2_O solvent system (1.5:1, *v/v*). Compounds **3** (56 mg) and **5** (65 mg) were isolated from subfraction F3 (6.4 g) via silica gel CC (CH_2_Cl_2_-MeOH, 100:0 to 0:100, gradient, *v/v*) and RP-C_18_ silica gel CC (MeOH–H_2_O, 2.5:1, *v/v*). Compound **2** (31 mg) was obtained from subfraction F4 (5.8 g) using silica gel CC eluted with CHCl_3_–MeOH (20:1 to 0:1, *v/v*), a Sephadex LH-20 column using the MeOH–water system (1:1, *v/v*), and silica gel CC with *n*-hexane–acetone (4:1 *v/v*). Compound **4** was obtained from subfraction F13 (5.4 g) using silica gel CC eluted with CHCl_3_–MeOH (20:1 to 0:1, *v/v*) and RP-C_18_ silica gel CC using a MeOH–H_2_O (2:1, *v/v*) solvent system. The purity of the compounds was checked via TLC (precoated Merck Kiesel gel 60 F_254_ plates and RP-18 F_254S_ plates) with various solvent system and visualized with 50% H_2_SO_4_. In comparison with previous data, compounds **1**–**5** were identified as sanggenofuran A (SA) [26], mulberrofuran D2 (MD2) [27], mulberrofuran D (MD) [28], mulberrofuran H (MH) [28], and morusalfuran B (MB) [29], respectively, by spectroscopic examination, including ^1^H and ^13^C-NMR. The chemical structures of the compounds are described in Figure 1.

### 4.4. PTP1B Inhibitory Assays

Inhibition of *p*NPP hydrolysis catalyzed by PTP1B was carried out by the method as described but with slight modification [30]. Ursolic acid was used as a positive control. The inhibitory activity exhibited by the tested compounds was expressed as the concentration capable of inhibiting 50% of the enzymatic activity (IC_50_) in μM unit.

### 4.5. Enzyme Kinetic Analysis

Among the tested compounds, most active compounds were subjected to kinetic analysis. The kinetic parameters were determined using Lineweaver-Burk double-reciprocal plots and the Dixon plot method at increasing substrate and compound concentrations [31,32,33]. All measurements were measured in triplicate, and Sigma Plot 12.0 (SPCC Inc., Chicago, IL, USA) was used to calculate the experimental parameters.

### 4.6. In Silico Docking Analysis

In silico docking analysis were performed using AutoDock 4.2 software [34]. X-ray crystallographic structures of PTP1B (PDB ID: 1T49) with its potent selective inhibitor compound **A** (3-(3,5-dibromo-4-hydroxy-benzoyl)-2-ethyl-benzofuran-6-sulfonic acid (4-sulfamoyl-phenyl)-amide) was obtained from RCSB Protein Data Bank at a 1.9 Å resolution [19]. Water molecules and inhibitors were removed from the structure using Discovery Studio 2017 R2 (Accelrys, San Diego, CA, USA). The 3D structure of the compounds and energy minimization was determined using Chem3D Pro 12.0 (CambridgeSoft, Cambridge, MA, USA) and their pKa values were computed at pH 7.0 using the MarvinSketch (ChemAxon, Budapest, Hungary). AutoDock 4.2 was used for docking simulations, and grid maps were generated using the AutoGrid program (grid box size of 126×126×126 had a default spacing of 0.375 Å). The docking protocol for rigid and flexible ligand docking comprised 10 independent genetic algorithms. The protocol was validated using the RMSD value. The selective catalytic inhibitor compound **C**, (3-({5-[(n-acetyl-3-{4-[(carboxycarbonyl)(2-carboxyphenyl)amino]-1-naphthyl}-l-alanyl)amino]pentyl}oxy)-2-naphthoic acid) and allosteric inhibitor compound **A** were used to compare interaction residues and dispositions [19,25]. Docking results were visualized and analyzed using PyMOL (v1.7.4, Schrödinger, LLC, Cambridge, MA, USA) and LigPlot.

### 4.7. Statistics

All data are presented as the mean ± standard deviation (SD) of triplicate samples of independent experiments. Statistical comparison between groups was performed using one-way ANOVA followed by Student’s t-tests (Systat Inc., Evanston, IL, USA).

## Figures and Tables

**Table 1:** Protein tyrosine phosphatase 1B (PTP1B) inhibitory activity of arylbenzofurans isolated from *Morus alba.*

Compounds	IC_50_ (μM) ^a^	Inhibition Type ^b^	*K*_i_ Value (μM) ^c^
SA	31.85 ± 2.98	‒	‒
MD2	3.11 ± 0.10	Noncompetitive	2.63
MD	11.61 ± 0.19	Mixed	4.79
MB	12.00 ± 0.75	Mixed	2.84
MH	53.47 ± 12.5	‒	‒
Ursolic acid ^d^	7.47 ± 1.24	‒	‒

^a^ The 50% inhibitory concentration (IC_50_) values (μM) were calculated from a log-dose inhibition curve and are expressed as the mean ± SD of triplicate experiments. ^b, c^ Inhibition type and inhibition constant were determined by Lineweaver-Burk and Dixon plots, respectively. ^d^ Positive control.

**Table 2:** In silico docking results of selected compounds.

Compound	Binding Energy (kcal/mol)	No. of H-Bonds	H-Bond Interacting Residues	Hydrophobic Interacting Residues
Compound **C** ^a^	−10.18	11	Ser216, Arg221, Ala217, Ile219, Gly220, Arg24, Arg254, Asp48	Tyr46, Cys215, Lys120, Thr263, Cln266, Val149, Met258, Gln262, Asp29, Arg24, Ser28
Compound **A** ^b^	−10.98	2	Asn193, Glu276	Ala189, Leu192, Phe196, Gly277, Lys279, Phe280, Ile281, Met282
MD2	−9.51	2	Lys197, Asn193	Phe280, Phe196, Leu192, Ala189, Glu200, Ser187, Glu276, Gly277
MD	−6.71	2	Gln266, Ala217	Asp48, Tyr46, Val49, Met258, Arg254, Gly259, Gln262, Arg24, Ile219, Gly220, Ser216
	−7.93	2	Ala189, Ser187	Asn193, Phe196, Gly277, Leu192, Lys279, Phe280, Glu276, Pro188
MB	−6.44	3	Gly183, Gly220, Ile219	Phe182, Gln266, Gln262, Asp181, Trp179, Glu115, Lys116, Lys120, Tyr46, Ser216, Ala217, Arg221
	−7.13	1	Lys197	Phe280, Asn193, Met282, Lys279, Ala189, Glu276, Gly277, Leu192, Ile281, Phe196, Glu200

^a,b^ Standard compounds for catalytic and allosteric inhibition, respectively.
